# Chronic kidney disease and mortality in fragility fracture patients: revisiting GFR thresholds

**DOI:** 10.1007/s41999-025-01286-w

**Published:** 2025-08-17

**Authors:** Joany Mariño, Paula Strittmatter, Maik Gollasch, Matthias Frank, Maximilian König

**Affiliations:** 1 Department of Internal Medicine B, University Medicine Greifswald, Greifswald, Germany; 2 German Centre for Cardiovascular Research (DZHK), Partner Site Greifswald, Greifswald, Germany; 3 Department of Internal Medicine D - Geriatrics, University Medicine Greifswald, Greifswald, Germany; 4 Geriatric Medicine Center, Kreiskrankenhaus Wolgast, Wolgast, Germany; 5 Department of Surgery, Kreiskrankenhaus Wolgast, Wolgast, Germany

**Keywords:** Frailty, Kidney function, eGFR, Age-adjusted, Mortality, Older adults, Fragility fracture, MDRD, CKD-EPI

## Abstract

**Purpose:**

Chronic kidney disease (CKD) is common in older adults. Still, its significance in patients with fragility fractures remains unclear, as does the clinical relevance of only mild-to-moderate CKD (GFR of 45–59 ml/min/1.73 m^2^) in older adults in general. We investigated how different eGFR categories (< 45, 45–59, and ≥ 60 ml/min/1.73 m^2^) are associated with mortality and functional outcomes in orthogeriatric patients.

**Methods:**

This retrospective cohort study included 453 consecutive patients admitted to an orthogeriatric unit from 2015 to 2023. Estimated glomerular filtration rate (eGFR) at admission was categorized into < 45, 45–59, and ≥ 60 ml/min/1.73 m^2^. Outcomes included mortality (via Kaplan–Meier curves, regression, and ROC analysis), in-hospital complications, and functional recovery. Mediation by frailty was also analyzed.

**Results:**

Patients had a mean age of 82.9 ± 6.8 years; 74.8% were female. CKD was prevalent: 52.4% had eGFR < 60, and 33.6% had eGFR < 45 ml/min/1.73 m^2^. Those with eGFR < 45 ml/min/1.73 m^2^ had worse baseline functional status, more comorbidities, and poorer recovery. The 45–59 group showed similar or better outcomes than those with eGFR ≥ 60 ml/min/1.73 m^2^, including mortality. Long-term all-cause mortality was significantly higher in the < 45 ml/min/1.73 m^2^ group (HR 1.77, 95% CI 1.25–2.51), but not in the 45–59 ml/min/1.73 m^2^ group (HR 1.01, 95% CI 0.64–1.58).

**Conclusion:**

In orthogeriatric patients, an eGFR < 45 ml/min/1.73 m^2^ identifies individuals at higher risk of poor outcomes. However, eGFR 45–59 is not associated with adverse prognosis and may not warrant classification as CKD in this context. A lower diagnostic threshold may better reflect clinical realities in this population.

**Supplementary Information:**

The online version contains supplementary material available at 10.1007/s41999-025-01286-w.

## Key summary points

**Aim:**

This study aims to provide a more nuanced understanding of chronic kidney disease (CKD) diagnostic thresholds and their implications for patient outcomes in orthogeriatric populations.

**Findings:**

This study found that reduced renal function is independently associated with mortality after fragility fractures. However, only patients with an eGFR < 45 ml/min/1.73 m^2^ had a significantly elevated long-term mortality risk, while those with eGFR 45–59 ml/min/1.73 m^2^ had outcomes similar to those with preserved renal function.

**Message:**

In orthogeriatric patients, an eGFR < 45 ml/min/1.73 m^2^ indicates a higher risk of poor outcomes. Meanwhile, eGFR 45–59 may not reflect adverse prognosis, suggesting that a lower CKD threshold could better align with clinical realities in this group.

**Supplementary Information:**

The online version contains supplementary material available at 10.1007/s41999-025-01286-w.

## Introduction

As life expectancy increases, identifying patient subgroups at heightened risk of adverse outcomes becomes increasingly important, particularly in older populations where chronological age alone is insufficient to capture individual health trajectories. In this context, chronic kidney disease (CKD) is particularly relevant, as it is strongly associated with adverse health events, including cardiovascular disease (CVD), fractures, and mortality [1]. Notably, CKD is one of the top five principal diagnoses linked to hospital readmissions, reflecting its significant burden on healthcare systems [2]. The prevalence of CKD is rising globally, particularly in older adults, affecting 50% or more of individuals in their eighties, partially due to the age-associated decline in glomerular filtration rate (GFR) [3, 4].

Not only the prevalence of CKD but also the incidence of osteoporotic fractures, particularly hip fractures, is increasing due to aging populations and lifestyle factors [5, 6]. Fragility fractures are among the leading causes of morbidity and mortality in older adults [7]. The so-called “Orthogeriatric Fracture Syndrome” needs to be explored in more detail as healthcare professionals (following the ongoing demographic change) are increasingly exposed to it [8]. The prevalence of CKD in hip fracture patients is very high [9]. While the association between CKD and fractures is established, the specific interplay between CKD and hip fracture outcomes remains understudied, particularly in multimorbid, frail, older populations [10]. CKD is thought to contribute to frailty through mechanisms such as inflammation, malnutrition, and muscle wasting, all of which increase the risk of falls and fractures [11]. Hence, investigating the common combination of CKD and osteoporotic fractures together is crucial to informing tailored interventions and improving outcomes in orthogeriatric patients.

Ongoing debates persist regarding the appropriateness of current CKD diagnostic thresholds in older individuals, as the physiological age-related decline in GFR may not always signify pathological disease [12, 13]. In particular, the clinical significance of an GFR between 45–60 mL/min/1.73 m^2^ (mild-to-moderate CKD) in older adults is disputed [12, 14–20], and it has been suggested that only GFR < 45 ml/min/1.73 m^2^ is associated with increased mortality risk [10]. Growing evidence suggests that older adults with stable GFR values between 45 and 59 ml/min/1.73 m^2^ often have outcomes comparable to those with GFR ≥ 60 ml/min/1.73 m^2^, challenging the clinical relevance of the 60 ml/min/1.73 m^2^ threshold [21–23]. Furthermore, it has been shown that an age-adapted criterion is more closely associated with the risk of morbidity and mortality than fixed threshold criteria [24, 25].

Most of the current evidence on the significance of GFR thresholds comes from large cohort studies, many of them focused on populations with above-average health (e.g. the MRC Trial of assessment and management of older people in the community) or specialized nephrological cohorts, where the average age is usually in the range of 65–75 years [13, 24, 26–28]. Therefore, there is a need for evidence coming from “real-world” geriatric populations to determine optimal diagnostic criteria and improve outcome prediction.

Here, we focus on older orthogeriatric patients and aim to investigate the clinical and prognostic significance of CKD through the following hypotheses:

1. Baseline characteristics and clinical trajectories do not differ significantly between patients with estimated GFR (eGFR) 45–59 and ≥ 60 ml/min/1.73 m^2^.
2. eGFR < 45 ml/min/1.73 m^2^ (but not eGFR 45–59) independently predicts (1-year and long-term) mortality and is associated with other adverse in-hospital outcomes in orthogeriatric patients, such as delirium, infections, and functional recovery.

By addressing these hypotheses, we strive to provide a more nuanced understanding of CKD diagnostic thresholds and their implications for patient outcomes in orthogeriatric populations and beyond. More specifically, we seek to better understand the “personality of the ortho-geriatric fracture patient” in order to improve patient-centered, holistic care.

## Methods

We performed a retrospective data analysis of consecutive patients admitted to the orthogeriatric unit at Wolgast District Hospital, Germany, between 2015 and 2023 (n = 571). All patients met the OPS 8-550 criteria for early geriatric rehabilitation. In general, included patients were aged 70 years or older, had sustained a fragility fracture, exhibited typical multimorbidity, had an Activities of Daily Living (ADL) score below 70, a positive geriatric screening, and demonstrated rehabilitation potential.

### Kidney function and mortality

This analysis used all records with kidney function measures available (n = 453). Patients who had been hospitalized in the orthogeriatric unit several times (n = 40) were only considered once at random. Serum creatinine was measured at admission (together with other routine laboratory parameters, including NTproBNP, C-reactive protein (CRP) and Vitamin D), and the estimated glomerular filtration rate (eGFR) was calculated using the MDRD [29] and CKD-EPI equations [30]. eGFR was grouped into < 45 ml/min/1.73 m^2^, 45–59 ml/min/1.73 m^2^, and ≥ 60 ml/min/1.73 m^2^. All analyses were primarily conducted using eGFR_MDRD_ and reported accordingly in the main manuscript. They were repeated using eGFR_CKD-EPI_, with selected results presented in the supplementary material.

One-year and long-term mortality was determined by linking clinical data from the electronic medical record and hospital discharge records with the civil registry using common personal identifiers (e.g. personal health number, name, sex, and date of birth).

### Covariables

Comorbidities were assessed using the updated Charlson comorbidity index (CCI) [31], generated from 12 comorbidities. For each patient, we retrieved all records to identify comorbidities. The presence of a comorbid condition was assigned to a patient when it was present in the index or previous admission records. Otherwise, the absence of the condition was assigned to the patient.

Frailty was assessed following the Frailty Index (FI) approach as described by Theou et al., assigning an FI between 0 and 1 to every patient [32]. Frailty was either used as a continuous variable or standardized to have a mean of 0 and a standard deviation of 1 (see Supplementary Methods Table 1 for a complete list of FI items).

On admission, all patients underwent a *Comprehensive Geriatric Assessment* (*CGA*), which evaluated the following aspects:

The *Activities of Daily Living* (*ADL*) considered were the presence or absence of urinary and/or fecal incontinence, help needed with grooming, toilet use, feeding, transfers (e.g. from chair to bed), walking, dressing, climbing stairs, and bathing. They were recorded both at admission and at discharge from the orthogeriatric unit. A higher score (max. 100) is associated with a greater likelihood of being able to live at home without problems [33].

*Handgrip Strength *(*HGS*) was measured with a handheld dynamometer. The maximum strength of the dominant hand was used in the analyses. Sex-specific cutoffs were used to define “low handgrip strength” (men: < 26 kg, women: < 17 kg) [34].

*Cognitive impairment* was established based on several established assessment tests by a neuropsychologist [35].

The *Tinetti test* is the most widely used clinical test for assessing a person’s static balance abilities and gait. The maximum achievable score is 28 [36]. The *Timed Up&Go *(*TUG*) is a commonly used screening test for mobility and falls. A faster time indicates a better gait performance (stand up, walk, turn, sit down) [37].

The *Geriatric Depression Scale* (*GDS*) is a simple, 15-item self-report instrument used to identify clinical depression among older adults. Higher scores indicate more symptoms, and a score of ≥ 5 suggests depression [38].

The German “Pflegegrad” system consists of five care levels, ranging from “Pflegegrad 1” (minimal care needs) to “Pflegegrad 5” (maximum care needs), with each grade reflecting the severity of a person’s physical, mental, and cognitive impairments.

### Statistical analyses

Participants’ characteristics were compared between strata using a t-test, one-way analysis of variance (ANOVA), Kruskal‒Wallis test, Wilcoxon rank sum test, Fisher’s test, or χ^2^ test, as appropriate. The primary outcomes were 1-year mortality and long-term all-cause mortality. The main exposure was kidney function, categorized based on estimated glomerular filtration rate (eGFR) into three groups: eGFR < 45 ml/min/1.73 m^2^, 45–59 ml/min/1.73 m^2^, and ≥ 60 ml/min/1.73 m^2^, corresponding to KDIGO stages ≤ G3b, G3a, and ≥ G2 [39].

#### 1-year mortality

One-year and long-term death rates per 100 PY with 95% confidence intervals were calculated. Logistic regression models were used to analyse the association between kidney function and 1-year mortality by estimating crude and adjusted odds ratios (OR) and corresponding 95% confidence intervals (CI) including a priori identified confounders of the relation between kidney function and mortality. The final adjusted models included the following variables: age at baseline (continuous), sex, the FI (standardized), fracture type and activities of daily living score (ADL) at discharge, and the CCI score. Effect modification by sex was investigated by including an interaction term between kidney function and sex to explore potential differences in the association between kidney function and mortality across sexes. Regression analyses excluded patients with missing data on covariates (n = 28).

#### Long-term all-cause mortality

Survival analysis was performed using July 2024 as the censoring date if still alive. Long-term death rates per 100 PY with 95% confidence intervals were calculated. We calculated Kaplan–Meier curves and mortality rates per 100 person-years (PY) with 95% confidence intervals, stratified by eGFR < 45, 45–59, and ≥ 60 ml/min/1.73 m^2^ at baseline. Cox proportional-hazards regression was used to assess the relationship between kidney function and the risk of death over the entire available observation period, adjusting for potential confounders. The following covariates were included in the model: age (continuous), sex, the FI (standardized), fracture type, activities of daily living score (ADL) at discharge, and comorbidity burden (CCI). The proportional hazards assumption was checked using Schoenfeld residuals. For variables that violated this assumption, time-varying effects were specified as interactions with natural cubic splines of follow-up time, allowing their Hazard Ratios (HR) to vary smoothly over time. HRs and 95% confidence intervals (CIs) were estimated for each covariate, and its significance was assessed using the Wald test.

#### Mediation analyses

We investigated the potential causal mediation between categories of impaired kidney function and mortality through frailty. Multivariable regressions were performed to estimate the effect of the exposure on the outcome after accounting for the mediator (reported as an adjusted OR with 95% CI). Substantial attenuation after accounting for the mediator was considered as suggesting the existence of a potential mediating pathway [40]. Moreover, we conducted a sensitivity analysis to evaluate whether adjusting for age and sex as confounders altered the relationships.

#### ROC curve analysis

To assess eGFR’s ability to predict long-term mortality, we performed a Receiver Operating Characteristic (ROC) curve analysis. The ROC curve plots sensitivity (true positive rate) against 1−specificity (false positive rate) across the range of possible eGFR values, providing a comprehensive evaluation of the biomarker’s discriminatory power. We calculated the Area Under the Curve (AUC) to quantify the biomarker’s ability to distinguish between survival and mortality outcomes. To determine an optimal classification threshold, we used Youden’s Index, which is defined as Sensitivity + Specificity − 1. This metric identifies the cut-off point that maximizes the trade-off between sensitivity and specificity. Additionally, we visualized the selected threshold by plotting sensitivity and specificity as functions of the biomarker value.

All analyses were performed using Stata SE 18.0 (StataCorp, College Station, TX) or R (version 4.4.1, R Core Team, 2024) using the packages survival, mediation, pROC, ggplot2, and ggbeeswarm [40–47]. The manuscript was prepared in compliance with the STrengthening the Reporting of Observational Studies in Epidemiology (STROBE) statement [48].

## Results

### Baseline characteristics

Of the 571 cases in this cohort, 453 unique patients with eGFR measurements taken on admission were included in this analysis (Supplementary Fig. 1). The mean patient age at baseline was 82.9 ± 6.8 years (range 62–98 years), with the majority being female (n = 339, 74.8%). As an orthogeriatric cohort, most patients were treated for proximal femur fractures (n = 344, 75.9%), while humerus fractures accounted for 15.7% of cases (n = 71). The remaining 8.4% had other orthogeriatric diagnoses, such as pelvic or vertebral fractures. Regarding comorbidities, dementia had been previously diagnosed in 16.4% of patients (n = 74), atrial fibrillation in 27.7% (n = 125), other cardiac diagnoses in 47.9% (n = 215), and diabetes mellitus in 32.1% (n = 145). The mean FI was 0.38 (range 0.12–0.66).

Kidney function impairment was highly prevalent, with 52.3% (n = 237/453) of patients having an estimated glomerular filtration rate (eGFR_MDRD_) below 60 ml/min/1.73 m^2^. Using an age-adjusted eGFR threshold for older adults (< 45 mL/min/1.73 m^2^), the prevalence remained substantial at 33.6% (n = 152). Using the CKD-EPI formula, 38.9% (n = 176) showed an eGFR_CKD-EPI_ < 45 mL/min. Table 1 provides a detailed summary of the cohort’s demographic and clinical characteristics at baseline.

**Table 1:** Baseline characteristics of the sample population, total and according to different eGFR_MDRD_ levels

	Total	eGFR < 45 ml/min/1.73 m^2^	eGFR 45–59 ml/min/1.73 m^2^	eGFR ≥ 60 ml/min/1.73 m^2^	p-value
	n = 453	n = 152 (33.6%)	n = 85 (18.8%)	n = 216 (47.7%)	
Sex, female	339 (74.8)	114 (75.0)	59 (69.4)	166 (76.9)	0.407
Age, years	82.90 ± 6.75	84.68 ± 5.94	82.89 ± 6.45	81.65 ± 7.15	0.001
Charlson comorbidity index (CCI)	7.16 ± 1.98	7.76 ± 1.99	7.01 ± 2.00	6.81 ± 1.89	< 0.001
Number of medications	9.24 ± 3.34	10.28 ± 3.22	9.07 ± 3.00	8.58 ± 3.40	< 0.001
Femoral fracture	344 (75.9)	115 (75.7)	63 (74.1)	166 (76.9)	0.859
Frailty Index	0.38 ± 0.09	0.42 ± 0.09	0.38 ± 0.07	0.36 ± 0.09	< 0.001
Heart disease^#^	215 (47.9)	96 (64)	42 (49.4)	77 (36.0)	< 0.001
Diabetes mellitus	145 (32.1)	64 (42.4)	24 (28.2)	57 (26.4)	0.004
Dementia	74 (16.4)	25 (16.6)	10 (11.8)	39 (18.2)	0.397
BMI, kg/m^2^	26.52 ± 5.33	26.86 ± 4.92	27.39 ± 5.57	25.89 ± 5.49	0.090
CRP, mg/L	95.1 (52.6; 137)	113.5 (69.8; 160)	84.2 (45.1; 132)	84 (8.1; 127)	< 0.001
25-OH Vit. D, μg/l	14.1 (7.0; 28.4)	15.1 (7.2; 32.7)	11.3 (7.0; 25.4)	15.1 (6.9; 28.3)	0.329
Hemoglobin, mmol/l	5.91 ± 1.00	5.69 ± 0.92	5.95 ± 1.08	5.90 ± 0.99	0.002
NT-proBNP, pg/mL	1406 (513; 3924)	3369 (1448; 7978)	1034 (409; 2604)	799 (335; 2128)	< 0.001
All cause mortality	78 (17.2)	35 (23.0)	12 (14.1)	31 (14.4)	0.067

Data are presented as mean ± standard deviation, number of observations (percentage), or median (IQR = 25th; 75th percentile). Number of missing values: CCI = 13, NT-proBNP = 328, Hemoglobin = 447, 25-OH Vit. D = 371, BMI = 373, CRP = 433, ADL Score = 28

*BMI* body mass index, *BNP* brain natriuretic protein, *CRP* C-reactive protein, *25-OH Vit. D* 25-hydroxy vitamin D, *eGFR* estimated glomerular filtration rate, #coronary artery disease/congestive heart failure/non-AF-arrhythmias/valvular cardiomyopathy, *MDRD* modification of diet in renal disease

Several comorbid conditions, including diabetes and cardiac diagnoses (i.e. congestive heart failure (CHF), coronary artery disease (CAD), valvular heart disease, and atrial fibrillation), were prevalent and showed a positive dose-dependent relationship with declining renal function (Table 1). Vitamin D levels were above-average in the eGFR_MDRD_ < 45 ml/min/1.73 m^2^ group. This may be explained by the fact that the proportion taking vitamin D supplements was highest in this group: 37.4% vs. only 19.4% (eGFR_MDRD_ 45–59 ml/min/1.73 m^2^) and 22.4% (eGFR ≥ 60 ml/min/1.73 m^2^), respectively (p = 0.029). CRP and BNP were by far the highest in the < 45 ml/min/1.73 m^2^ group and similar in the other two groups.

### Comprehensive geriatric assessment (CGA)

**Fig. 1:**
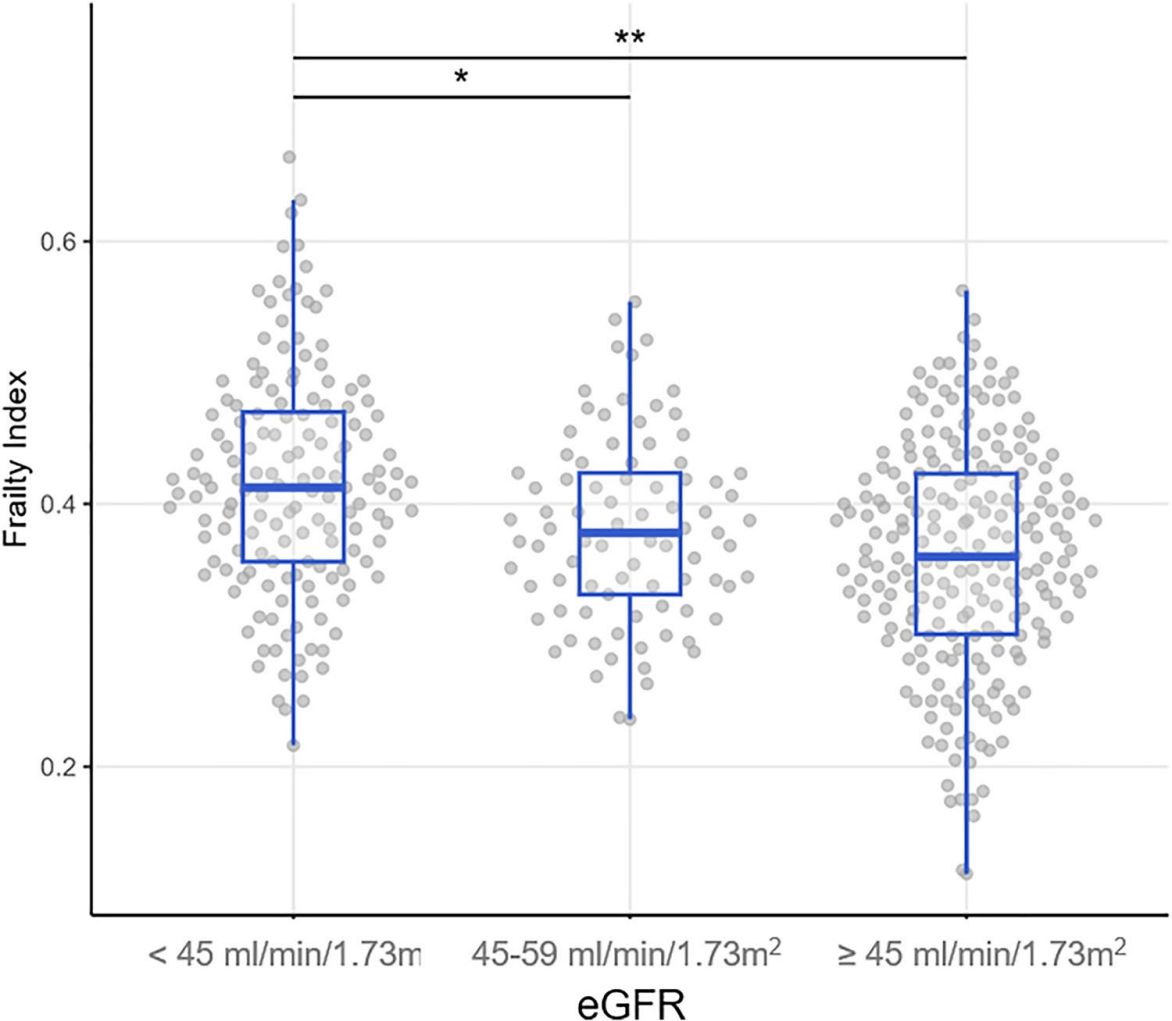
Frailty Index distribution by eGFR_MDRD_ category. *p-value = 0.005, **p-value < 0.001

The results of the CGA are shown in Supplementary Table 1. Overall, severe impairments in ADL were highly prevalent. 29.8% had elevated depression screening scores, 61.4% had possible sarcopenia (low handgrip strength), 41.3% showed evidence of cognitive impairment, and almost all had mobility impairment in the TUG. Notably, when stratified by eGFR_MDRD_ into < 45, 45–59, and ≥ 60 ml/min/1.73 m^2^, those with eGFR < 45 ml/min/1.73 m^2^ showed the worst performance status across all domains of the CGA. In contrast, the 45–59 ml/min/1.73 m^2^ group had functional abilities (e.g. ADL) similar or slightly better than those with eGFR ≥ 60 ml/min/1.73 m^2^. The mean FI was similar in patients with eGFR ≥ 60 and 45–59 ml/min/1.73 m^2^, but significantly higher in those with eGFR < 45 ml/min/1.73 m^2^ compared to both other groups (p = 0.005 and p < 0.001, respectively) (Fig. 1).

### The in-hospital phase

Patients with more severe kidney function impairment (eGFR_MDRD_ < 45 ml/min/1.73 m^2^) tended to have higher rates of postoperative ICU admission, delirium and less functional recovery (delta ADL) than patients with preserved kidney function (Supplementary Table 2). However, they did not exhibit higher rates of intercurrent infection, longer hospital stays, or significantly increased in-hospital mortality compared with patients with an eGFR of 45–59 ml/min/1.73 m^2^ or ≥ 60 ml/min/1.73 m^2^ (Supplementary Table 2). Interestingly, patients in the eGFR 45–59 ml/min/1.73 m^2^ group did not have worse outcomes than those with eGFR ≥ 60 ml/min/1.73 m^2^, and even showed a tendency toward better outcomes on certain measures (i.e. delirium, intrahospital death, and delta ADL).

### Patient characteristics at discharge and post-hospital care

At discharge, patients with eGFR_MDRD_ < 45 ml/min/1.73 m^2^ not only showed less functional recovery (delta ADL) but also had higher chances of new nursing home placement (Supplementary Table 3). Again, the intermediate category (eGFR 45–59 ml/min/1.73 m^2^) was not significantly different from those with eGFR ≥ 60 ml/min/1.73 m^2^, or even slightly better off.

Notably, patterns did not change when using the CKD-EPI equation instead of the MDRD.

#### 1-year mortality

In the first year after the index fracture, 78 out of 453 patients (17.2%) died (14.5% women and 25.4% men, p = 0.007). Overall, only a lower ADL score at discharge was associated with increased odds of death. Interaction analyses showed that the impact of eGFR < 45 ml/min/1.73 m^2^ compared to ≥ 60 ml/min/1.73 m^2^ on 1-year mortality was negative in women (OR 2.36, 95% CI 1.05–5.32, p = 0.039), while there was no evidence for the same effect in men (OR 0.77 (95% CI 0.21–2.87) (Table 2). In both women and men, the intermediate category (eGFR 45–59 ml/min/1.73 m^2^) was clearly not associated with increased mortality compared to eGFR > 60 ml/min/1.73 m^2^.

**Table 2:** Predictors of 1-year mortality (multivariable logistic regression), N = 425

	OR	95% CI	p-value
Age, years	1.00	0.95, 1.05	0.914
Female sex	1.00 (Ref.)	–	–
Male sex	2.06	0.72, 5.31	0.178
eGFR_MDRD_ categories (men)			
≥ 60 ml/min	1.00 (Ref.)	–	–
45–59 ml/min	1.41	0.39, 5.17	0.602
< 45 ml/min	0.77	0.21, 2.87	0.699
eGFR_MDRD_ categories (women)			
≥ 60 ml/min	1.00 (Ref.)	–	–
45–59 ml/min	1.20	0.35, 4.07	0.775
< 45 ml/min	2.36	1.05, 5.32	0.039
ADL score	0.97	0.96, 0.99	< 0.001
Frailty Index (standardized)	1.27	0.82, 1.96	0.287
CCI score	1.15	0.97, 1.37	0.108
Fracture type			
Femur	1.00 (Ref.)	–	–
Humerus	0.38	0.12, 1.20	0.099
Other	0.40	0.09, 1.87	0.244

*Ref*. reference category for categorical variables, *OR* odds ratio, *CI* confidence interval, *eGFR* estimated glomerular filtration rate (unit: ml/min/1.73 m^2^), *MDRD* modification of diet in renal disease

#### Long-term all-cause mortality

**Fig. 2:**
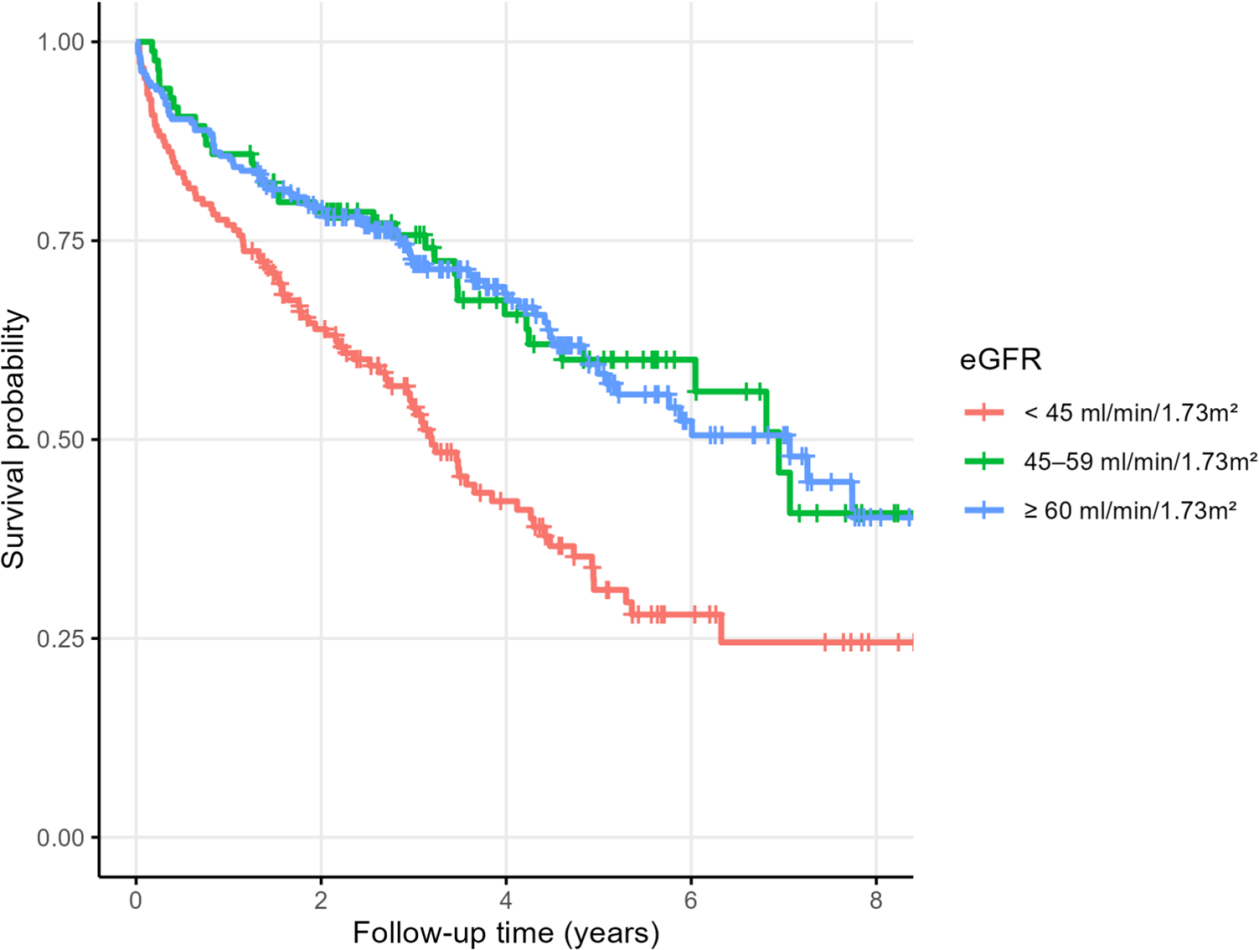
Kaplan–Meier curves showing survival probability according to eGFR_MDRD_ levels. *eGFR* estimated glomerular filtration rate, *MDRD* modification of diet in renal disease

During the total available follow-up (median FU duration 3.3 years, max. 8.7 years) 203 out of the initial 453 (44.8%) individuals died. Kaplan–Meier curves (Fig. 2) revealed a clear divergence in survival probability according to renal function, stratified into eGFR_MDRD_ < 45 ml/min/1.73 m^2^ vs. 45–59 vs. ≥ 60 ml/min/1.73 m^2^. The death rate was 21.79 (95%-CI 17.72–26.79) per 100 PY in participants with eGFR < 45 ml/min, compared to 10.44 (95% CI 7.42–14.58) per 100 PY in those with eGFR 45–59 ml/min/1.73 m^2^ and 10.72 (95% CI 8.61–13.35) per 100 PY when eGFR ≥ 60 ml/min. Over the total follow-up, impaired kidney function (eGFR < 45 ml/min) was associated with increased mortality (RR 2.03, 95% CI 1.50–2.75, p < 0.001).

**Fig. 3:**
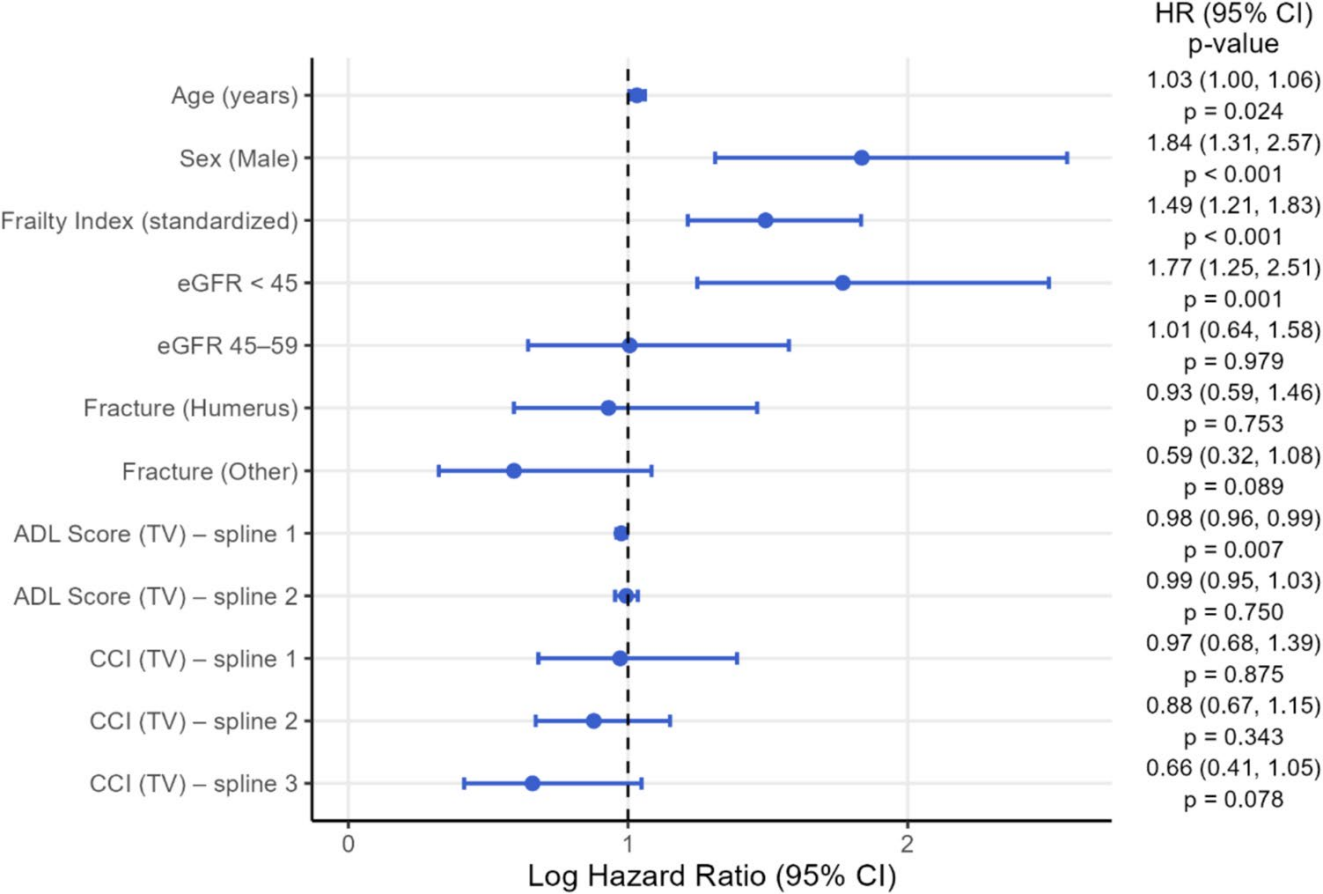
Forest plot showing the estimates of the Cox regression model for long-term all-cause mortality (log HR, 95% CI). *ADL* activities of daily living, *eGFR* estimated glomerular filtration rate (unit: ml/min/1.73 m^2^), *ADL* activities of daily living, *HR* hazard ratio, *CI* confidence interval, *CCI* Charlson Comorbidity Index, *TV* time varying

The likelihood-ratio test provided no evidence of interaction by sex (p = 0.8426). Multivariable-adjusted analysis confirmed that eGFR_MDRD_ < 45 ml/min was independently associated with increased mortality (HR 1.77, 95% CI 1.25–2.51, p < 0.001) **(**Fig. 3**)**, whereas eGFR 45–59 ml/min/1.73 m^2^ was consistently not associated with increased long-term mortality (HR 1.01, 95% CI 0.64–1.58, p = 0.979).

The results of the survival analyses did not differ significantly when eGFR was calculated using CKD-EPI instead of MDRD (see Supplementary Figs. 5 and 6).

#### ROC curve analysis

**Fig. 4:**
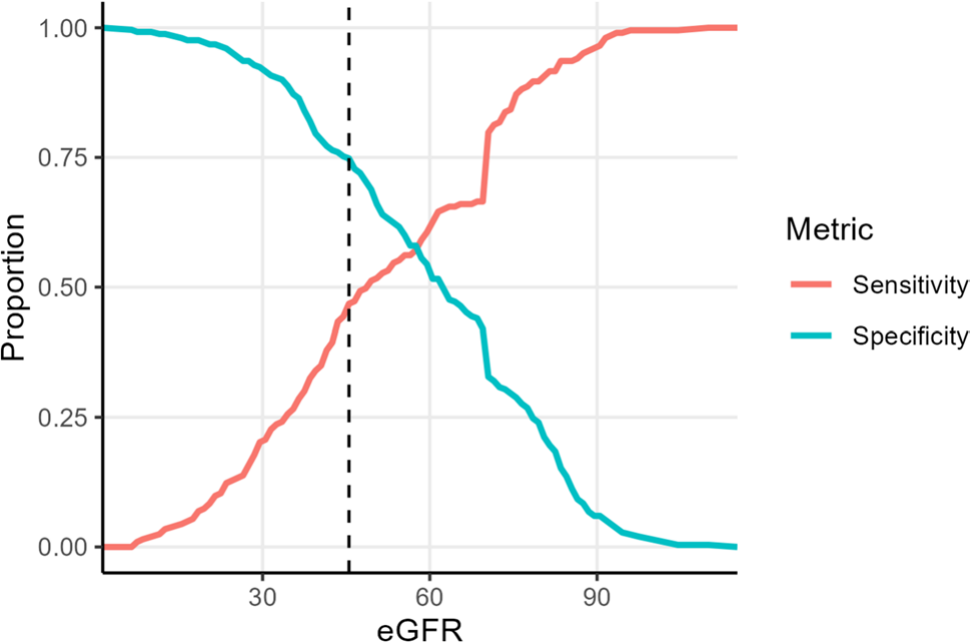
Sensitivity and specificity as a function of eGFR values. The eGFR threshold that maximizes the difference between the true positive rate (sensitivity) and the false positive rate (1- specificity) is shown by the dashed line (eGFR = 45.5 ml/min/1.73 m^2^). *eGFR* estimated glomerular filtration rate

The ROC analysis yielded an AUC of 0.62 (Supplementary Fig. 8), indicating that the eGFR_MDRD_ had weak to moderate predictive ability for mortality. Using Youden’s Index, the optimal threshold was identified at 45.5 ml/min/1.73 m^2^, supporting our initial hypothesis. At this threshold, sensitivity was 46.8%, meaning a considerable proportion of deceased individuals were misclassified as survivors. However, the specificity of 74.8% suggested that the eGFR_MDRD_ was relatively effective at correctly identifying survivors (Fig. 4). Analysis using eGFR estimated through the CKD-EPI equation gave similar results (Supplementary Figs. 9 and 10).

#### Mediation analyses

The association between renal impairment and death including frailty as a mediator (unadjusted) was significant when comparing eGFR_MDRD_ ≥ 60 vs. < 45 (OR 1.25 [1.13—1.38], p < 0.001) or 45–59 vs. < 45 (OR 1.22 [1.07–1.4], p < 0.001) (Supplementary Table 6 and Supplementary Fig. 7). However, the low proportion mediated (43% and 30%, respectively) and the significant direct effect between CKD and death (OR 1.14 [1.03–1.27], p < 0.001; and 1.15 [1.01–1.31], p = 0.04) suggested only partial mediation. On the contrary, the association between eGFR ≥ 60 vs. 45–59 ml/min/1.73 m^2^ was not significant (OR 1.02 [0.9–1.15], p = 0.722), suggesting that partial mediation through frailty may only occur when eGFR < 45 ml/min/1.73 m^2^. Sensitivity analysis adjusting for age and sex did not change these findings significantly (Supplementary Table 7). Overall, these findings indicated that frailty may partially mediate the association between more severe CKD and mortality. In contrast, eGFR 45–59 ml/min/1.73 m^2^ may not be associated with a worse prognosis compared to ≥ 60 ml/min/1.73 m^2^ once frailty is accounted for.

## Discussion

The results of this study demonstrate an independent association between reduced renal function and mortality following fragility fractures. However, upon closer scrutiny, only those with an eGFR < 45 ml/min/1.73 m^2^ faced a significantly elevated risk, experiencing an approximately two-fold increase in long-term mortality. In contrast, patients with an eGFR of 45–59 ml/min/1.73 m^2^ exhibited similar clinical characteristics and prognosis (HR 1.01, 95% CI 0.64–1.58) to those with an eGFR ≥ 60 ml/min, suggesting that mild-to-moderate renal impairment may not substantially impact mortality risk in this population.

### Limitations and strengths

Our results should be considered in light of a few limitations. First, our analysis are based solely on hospital data from the index event and vital status (date of death, if dead) from the civil registry. We lacked details on the cause of death, kidney function trajectories or other intercurrent diseases. While it seems reasonable to attribute deaths occurring within the first 12 months to the index diagnosis (i.e. fragility fracture) and baseline kidney function, the strength of this association likely diminishes with time.

Second, direct measurements of kidney function (i.e., measured GFR) were not available. Instead, we used eGFR based on the MDRD and CKD-EPI equations, which, although widely used in clinical practice, have known limitations in terms of accuracy, precision, and systematic bias. This is particularly relevant in older populations, due to age-related changes in muscle mass and creatinine generation [49]. Nonetheless, previous research has shown that, while all estimation equations tend to overestimate GFR below 60 ml/min/1.73 m^2^ in older adults, the MDRD and CKD-EPI equations retain similar and reasonable accuracy in this range [50, 51]. The need for both accurate GFR measurement or estimation and better risk stratification through potentially age-adjusted thresholds is widely accepted [39, 52, 53]. However, measured GFR is unlikely to become routinely available in the near future, and Cystatin C has not yet been widely adopted in clinical practice. Consequently, creatinine-based eGFR remains the most practical and commonly used method—making it essential for clinicians to understand both its value and limitations.

Third, we relied on a single measurement of creatinine, which is a typical limitation of epidemiological studies. Moreover, we lacked albuminuria measurements that could have provided further insights into renal function. Consequently, we cannot exclude the possibility of acute kidney injury (AKI), as factors such as trauma and hypotension (e.g., due to blood loss or sepsis) can contribute to its development in this clinical context. In such cases, “unspecified renal failure” may be the most appropriate classification. This diagnostic ambiguity—whether renal dysfunction is acute, chronic, or both—is common in clinical practice and reflects the inherent complexity of renal dysfunction in older adults.

Regarding frailty, the FI range in our study (0.12–0.66) closely aligns with that reported in the original studies by Rockwood et al. [32]. Moreover, there was a dose-dependent association between frailty and mortality. Though, the median FI of 0.39 was very high, with fewer than 10% having an FI below 0.25 indicating that over 90% of the cohort were frail. The high prevalence of frailty in our cohort introduces potential selection bias and may limit the generalizability of our findings to healthier or more heterogeneous populations. This overrepresentation of frailty also raises the possibility of spectrum bias, where the observed associations may not accurately reflect those seen in populations with a broader distribution of frailty.

Of course, the fact that this analysis only included people with fragility fractures limits the generalizability to populations of older adults without fractures.

### Age-adapted thresholds

Our study provides real-world evidence supporting the use of an age-adapted threshold for CKD classification based on eGFR in very old adults. We found that an eGFR < 45 ml/min/1.73 m^2^ was associated with significantly increased mortality (HR 1.77, 95% CI 1.25–2.51), compared to individuals with eGFR ≥ 60 ml/min/1.73 m^2^. In contrast, mortality was not increased in individuals with mild-to-moderate CKD (eGFR 45–59 ml/min/1.73 m^2^, adjusted HR 1.01, 95% CI 0.64–1.58).

According to our results, in this very old patient population, a mild-to-moderate CKD (eGFR 45–59 ml/min/1.73 m^2^) captures individuals who are at least not sicker and not more at risk than those with a preserved kidney function (eGFR ≥ 60 ml/min), and possibly even healthier. For this reason, we advocate for an age-adjusted diagnostic cut-off value for CKD in older adults, setting the threshold at eGFR < 45 ml/min/1.73 m^2^, as supported by ROC analysis, where the optimal value for mortality prediction was 45.5 ml/min/1.73 m^2^.

Our results align with previous findings. Cross-sectional analysis from the Berlin Aging Study II showed that an eGFR < 45 ml/min/1.73 m^2^—but not < 60 ml/min/1.73 m^2^—was associated with a higher likelihood of functional impairment (even if only in the mobility domain) [22], although the cohort was notably younger (mean age 68.6 ± 3.6 years). In contrast, our study population represents a truly geriatric cohort, representative of the patient spectrum in orthogeriatric units in Germany [54], providing a clearer and more robust signal. Another recent study has likewise recommended lower, age-specific thresholds for defining CKD in older adults (e.g. 50, 40, or even 30 ml/min/1.73 m^2^) [13].

While a single threshold may be more favorable for awareness and simplicity, our results favor the adoption of a lower cut-off in older populations. An age-adapted definition reduces the prevalence of CKD by up to 50% among old and very old individuals, and supports a more personalized, clinically relevant approach [55].

### Why do older adults with reduced eGFR < 45 ml/min/1.73 m^2^ have a poor prognosis while those with eGFR 45–59 ml/min/1.73 m^2^ do not?

Patients with an eGFR < 45 ml/min/1.73 m^2^ had the most unfavourable clinical profiles across all care phases—from admission, through perioperative and further inpatient course to discharge. These individuals exhibited a higher burden of cardio-metabolic comorbidities, elevated inflammatory markers (CRP), higher NT-proBNP levels, reduced handgrip strength, more polypharmacy, higher FI, and lowest recovery in ADL.

While CRP typically rises after fragility fractures due to fracture trauma and surgical stress, the elevation tended to be disproportionately high in patients with eGFR < 45, compared to 45–59 and ≥ 60 ml/min/1.73 m^2^, indicating excessive systemic inflammation. These patients also had a higher prevalence of conditions linked to chronic inflammation—such as low hand-grip strength indicative of sarcopenia, diabetes and cardiovascular disease—a pattern often referred to as “inflammaging” [56–58].

Increased NT-proBNP levels in this group may be attributed to both worse kidney function [59] and more (and probably more severe) underlying cardiac dysfunctions (e.g., heart failure, atrial fibrillation), which may contribute and partially explain the excess mortality. However, in sensitivity analysis, heart disease did not account for the observed association between kidney function and mortality (Supplementary Table 5).

Although often not statistically significant, the group with mild-to-moderate renal impairment (eGFR 45–59 ml/min) showed the most favorable overall profile— e.g. least anemic, best ADL score (Supplementary Table 1). This may be explained by the physiological basis of creatinine-based eGFR [60]. Serum creatinine is both a marker of renal function and muscle mass [61]. eGFR overestimates true GFR in individuals with low muscle mass (e.g. cachexia/sarcopenia), and underestimates it in individuals with high muscle mass [62, 63]. Therefore, a preserved eGFR in very old adults may reflect reduced creatinine production due to sarcopenia rather than true renal health—an unfavorable prognostic indicator. In contrast, individuals with an eGFR of 45–59 ml/min/1.73 m^2^ may be relatively muscle-healthy, explaining their better outcomes. This further supports the lack of excess risk in this group and highlights the potential value of age- and context-specific interpretation of eGFR.

In this vein, for 1-year mortality after a fragility fracture, a statistically significant association could only be demonstrated in women. This may be due to the male subgroup being too small (n = 114), but it is also possible that a true biological cause underlies the finding, as creatinine-based eGFR in older men—with and without sarcopenia—leads to different and potentially biased patterns compared to women [61].

### Is reduced kidney function a marker of poor health or a direct contributor to mortality?

As expected, individuals with eGFR < 45 ml/min/1.73 m^2^ exhibited significantly compromised clinical profiles across multiple physiological domains, prompting the question of whether underlying frailty (an integrative construct encompassing comorbidity, functional abilities, and bio-psychosocial factors) may explain this group's increased risk of adverse events and mortality. In our regression analysis, even after adjusting for frailty, age, sex, pre-fracture comorbidity burden, and activities of daily living, reduced kidney function remained significantly associated with mortality. This suggests that CKD exerts an independent effect on mortality risk beyond the influence of comorbidity and frailty. Further, mediation analysis revealed that frailty accounted for approximately 34% of the effect of CKD on mortality. In other words, while frailty does play a role in the pathway from CKD to mortality, the remaining 66% were attributable to other factors. This suggests that although frailty plays a meaningful role in the pathway linking CKD to mortality, other contributors—such as specific comorbidity patterns [64], subclinical electrolyte disturbances (in calcium-phosphate or electrolyte homeostasis [39]), or the well-established CKD–cardiovascular disease axis [65]—may be equally or more important. Our data support this interpretation, as individuals with reduced eGFR also had higher markers of cardiac dysfunction and systemic inflammation.

These findings are in line with prior studies. For instance, Fisher et al. showed a stepwise increase in risk of adverse outcomes with worsening kidney function in hip fracture patients, particularly when combined with comorbidities [9]. Similarly, a recent meta-analysis of observational studies confirmed the association between hip fracture and an increased risk of cardiovascular events. In this sense, CKD could be an interesting factor to be considered in managing hip fracture patients to improve the prognosis following hip fractures [66, 67].

It is also noteworthy that frailty can be measured using various models—including the clinical model, the cumulative deficit model, and the physical frailty phenotype (Fried) [32, 68]—and it remains possible that an alternative measure might better capture the mediating effect in this population. Future studies should explore these pathways further.

Our approach of studying the common combination of CKD and osteoporotic fractures together is innovative and clinically relevant, as these conditions never occur in isolation. Not least, the findings of this study underscore the compounded health challenges faced by this very old orthogeriatric population, with CKD acting as a significant factor associated with adverse outcomes.

## Conclusions

In very old adults, impaired kidney function below 45 ml/min/1.73 m^2^ (estimated by the MDRD and CKD-EPI equations) is independently associated with significantly higher mortality, even after adjusting for sex, age, comorbidities, activities of daily living, and frailty. In contrast, mild-to-moderate renal impairment does not appear to increase mortality risk in this frail, older population.

## Supplementary Information

Below is the link to the electronic supplementary material.

Supplementary file1 (DOCX 533 KB)

## Data Availability

Due to concerns for participant privacy, data are available only upon reasonable request from the corresponding author.
