# Supplementary material for: Chronic kidney disease and mortality in fragility fracture patients: revisiting GFR thresholds
Source: Eur Geriatr Med. 2025 Aug 17;16(6):2221–33. doi: 10.1007/s41999-025-01286-w (PMC12743717; doi:10.1007/s41999-025-01286-w)
Supplement: Supplementary file 1 — Supplementary file1 (DOCX 533 KB) [file 41999_2025_1286_MOESM1_ESM.docx]

**Supplementary Tables and Figures**

**Chronic Kidney Disease and Mortality in Fragility Fracture Patients: Revisiting GFR Thresholds**

Joany Mariño^1,2#^, Paula Strittmatter^3#^, Maik Gollasch^3,4^, Matthias Frank^3,5^, Maximilian König^3,4^

^1^Department of Internal Medicine B, University Medicine Greifswald, Germany

^2^German Centre for Cardiovascular Research (DZHK), partner site Greifswald, Greifswald, Germany.

^3^Department of Internal Medicine D - Geriatrics, University Medicine Greifswald, Germany

^4^Geriatric Medicine Center, Kreiskrankenhaus Wolgast, Wolgast, Germany

^5^Department of Surgery, Kreiskrankenhaus Wolgast, Wolgast, Germany

^#^Joany Mariño and Paula Strittmatter contributed equally to this article.


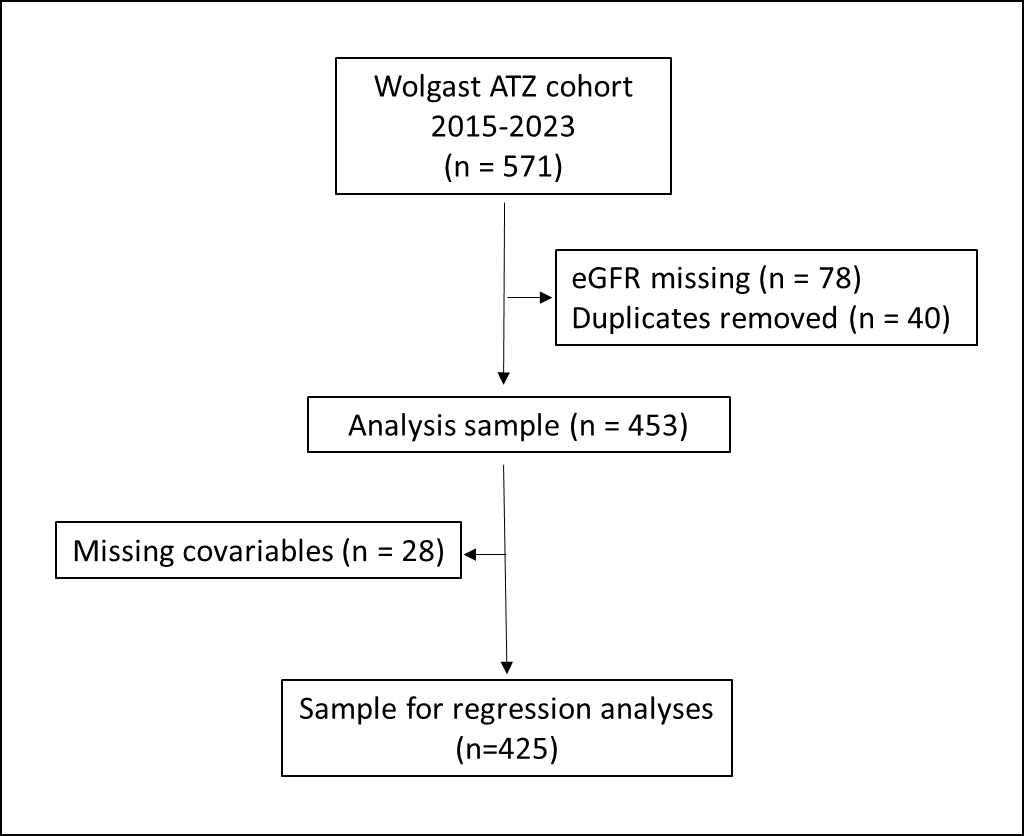


´

**Supplementary Figure 1.** Study Flow-chart; Abbreviations: ATZ = Alterstraumatologisches Zentrum, eGFR = estimated glomerular filtration rate

**Supplementary Methods Table 1.** Frailty Index items

|  | Item | Coding | Missing  N(%) | Health problem | Not too similar to another variable | Not too rare or too common | Age-associated | Not too highly correlated with other variables |
| --- | --- | --- | --- | --- | --- | --- | --- | --- |
| 1 | **COPD** | 0, no deficit  1, deficit | 3(0,6%) | Yes | Yes | Yes | Yes | Yes |
| 2 | **Diabetes**  **Mellitus** | 0, no deficit  1, deficit | 1 (0.2%) | Yes | Yes | Yes | Yes | Yes |
| 3 | **Stroke** | 0, no deficit  1, deficit | 44 (9.9%) | Yes | Yes | Yes | Yes | Yes |
| 4 | **Congestive heart failure** | 0, no deficit  1, deficit | 1 (0.2%) | Yes | Yes | Yes | Yes | Yes |
| 5 | **Coronary Artery Disease** | 0, no deficit  1, deficit | 1 (0.2%) | Yes | Yes | Yes | Yes | Yes |
| 6 | **Cancer** | 0, no deficit  1, deficit | 1 (0.2%) | Yes | Yes | Yes | Yes | Yes |
| 7 | **Osteoporosis** | 0, no deficit  1, deficit | 0 (0.0%) | Yes | Yes | Yes | Yes | Yes |
| 8 | **Depression** | 0, no deficit  0.5, half deficit (5-8 points)  1, deficit (>=9 points) | 101 (22.3%) | Yes | Yes | Yes | Yes | Yes |
| 9 | **Cognition score** | <10=1, 11-17=0.75, 18-20=0.5, 21-24=0.25, >24=0 | 90 (19.9%) | Yes | Yes | Yes | Yes | Yes |
| 10 | **Body mass index** | 0, no deficit  0.5, half deficit  1, deficit | 80 (17.7%) | Yes | Yes | Yes | Yes | Yes |
| 11 | **Clock drawing test** | 0, no deficit  1, deficit | 125 (27.6%) | Yes | Yes | Yes | Yes | Yes |
| 12 | **Independent walking** | 0, no deficit  0.5, wheelchair/walker  1, no walking | 24 (5.3%) | Yes | Yes | Yes | Yes | Yes |
| 13 | **Mobility (Tinetti Test)** | 0, no deficit  1, deficit (>20sec) | 25 (5.5%) | Yes | Yes | Yes | Yes | Yes |
| 14 | **Atrial fibrillation** | 0, no deficit  1, deficit | 1 (0.2%) | Yes | Yes | Yes | Yes | Yes |
| 15 | **Polypharmacy** | 0, (1-4 drugs)  0.5, (5-9 drugs)  1, (>=10 drugs) | 19 (4.2%) | Yes | Yes | Yes | Yes | Yes |
| 16 | **CKD (eGFR < 60 ml/min)** | 0, no deficit  1, deficit | 0 (0%) | Yes | Yes | Yes | Yes | Yes |
| 17 | **Dysnatremia** | 0, no deficit  1, deficit | 5 (1.1%) | Yes | Yes | Yes | Yes | Yes |
| 18 | **Dizziness** | 0, no deficit  1, deficit | 0 (0.0%) | Yes | Yes | Yes | Yes | Yes |
| 19 | **Parkinson’s disease** | 0, no deficit  1, deficit | 3 (0.7%) | Yes | Yes | Yes | Yes | Yes |
| 20 | **Hypocalcemia** | 0, no deficit  1, deficit | 26 (5.7%) | Yes | Yes | Yes | Yes | Yes |
| 21 | **Vitamin D deficiency** | 0, no deficit (>30)  0.5, half deficit (10-30)  1, deficit (<10) | 82 (18.1%) | Yes | Yes | Yes | Yes | Yes |
| 22 | **Hypo/Hyperthyreosis** | 0, no deficit (0.4-4)  0.5, half deficit (4-9)  1, deficit (< 0.4/>=10) | 125 (27.6%) | Yes | Yes | Yes | Yes | Yes |
| 23 | **Housing situation** | 0, at home  0.5, assisted living  1, Nursing home | 12 (2.7%) | Yes | Yes | Yes | Yes | Yes |
| 24 | **Rheumatism** | 0, no deficit  1, deficit | 0 (0.0%) | Yes | Yes | Yes | Yes | Yes |
| 25 | **Care level** | 0, no care level  0.25, level 1  0.5, level 2  0.75, level 3  1, level >= 4 | 28 (6.2%) | Yes | Yes | Yes | Yes | Yes |
| 26 | **Abnormal urinary status** | 0, no  1, yes | 4 (0.9%) | Yes | Yes | Yes | Yes | Yes |
| 27 | **Hemiplegia** | 0, no deficit  1, deficit | 0 (0.0%) | Yes | Yes | Yes | Yes | Yes |
| 28 | **Multimorbidity** | <5= 0, 5-6=0.25, 7-9= 0.5 0.75, >=10=1 | 0 (0.0%) | Yes | Yes | Yes | Yes | Yes |
| 29-38 | **Activities of daily living:**  **Feeding**  **Personal hygiene**  **Bathing**  **Dressing**  **Bowel control**  **Bladder control**  **Toilet use**  **Transfers (bed to chair and back)**  **Mobility (on level surfaces)**  **Stair climbing** | Together:  100-96=0, 95-91=0.5, 90-86=1, 85-81=1.5, 80-76=2, 75-71=2.5, 70-66=3, 65-61=3.5, etc. | 0 (0.0%) | Yes | Yes | Yes | Yes | Yes |
| 39 | **Valvular heart disease** | 0, no deficit  1, deficit | 2 (0.4%) | Yes | Yes | Yes | Yes | Yes |
| 40 | **Dementia** | 0, no deficit  1, deficit | 3 (0.7%) | Yes | Yes | Yes | Yes | Yes |

**Supplementary Table 1**. Results of the comprehensive geriatric assessment at admission.

|  | **Total** | **eGFR_MDRD_ < 45 ml/min/1.73m^2^** | **eGFR_MDRD_ 45-59 ml/min/1.73m^2^** | **eGFR_MDRD_ ≥ 60 ml/min/1.73m^2^** | **N** | ***p-value*** |
| --- | --- | --- | --- | --- | --- | --- |
| **ADL** | 35 (20;45) | 30 (20;40) | 40 (25;50) | 35 (25;45) | 444 | 0.006 |
| **GDS ≥ 5** | 105 (29.8 %) | 33 (27.5 %) | 21 (32.3 %) | 35 (30.5) | 352 | 0.763 |
| **Low handgrip strength** | 175(61.4 %) | 61(64.9 %) | 30(57.7 %) | 84(60.4 %) | 285 | 0.657 |
| **TUG** | 24(13;40) | 25 (15;45) | 22 (13.5;29.5) | 21 (12;45) | 133 | 0.458 |
| **Tinetti** | 3 (1;12) | 2 (1;8,5) | 4 (1;13) | 3 (1;12) | 436 | 0.292 |
| **Cognitive impairment** | 150 (41.3 %) | 62(50.8 %) | 25 (37.3 %) | 63 (36.1 %) | 363 | 0.032 |
| **Care level** | 1(0;2) | 1,5(0;2) | 0(0;2) | 0 (0;2) | 425 | 0.045 |

***Notes and abbreviations:*** Data are presented as mean ± standard deviation (SD), number of observations (percentage), or median (IQR = 25th;75th percentile). ADL = Activities of daily living, GDS = Geriatric depression scale, TUG = Timed Up&Go (test), eGFR = estimated glomerular filtration rate, MDRD = Modification of Diet in Renal Disease.

**Supplementary Table 2.** Participant characteristics during the in-hospital phase.

|  | **Total** | **eGFR_MDRD_ < 45 ml/min/1.73m^2^** | **eGFR_MDRD_ 45-59 ml/min/1.73m^2^** | **eGFR_MDRD_ ≥ 60 ml/min/1.73m^2^** | **N** | ***p-value*** |
| --- | --- | --- | --- | --- | --- | --- |
| **LOS, days** | 20 (18;26) | 21 (18;26) | 20 (17;28) | 20 (18;26) | 453 | 0.970 |
| **Delirium** | 92 (20.5 %) | 31 (29.7 %) | 12 (14.3 %) | 49 (22.9 %) | 448 | 0.254 |
| **Infections** | 100 (22.3 %) | 30 (19.9 %) | 18 (21.2 %) | 52 (24.4 %) | 449 | 0.569 |
| **ICU** | 200 (45.2 %) | 79 (52.0 %) | 35 (42.2 %) | 86 (41.4 %) | 443 | 0.112 |
| **Intrahospital death** | 14 (3.3 %) | 6 (4.1 %) | 1 (1.2 %) | 7 (3.3 %) | 446 | 0.487 |
| **Delta ADL** | 29.7± 20.4 | 28.8±20.4 | 32.4±20.7 | 29.3±21.3 | 425 | 0.421 |

***Notes and abbreviations:*** Data are presented as mean ± standard deviation (SD), number of observations (percentage), or median (IQR = 25^th^;75th percentile). ADL = activities of daily living, Delta ADL: functional recovery regarding activities of daily living (ADL at discharge minus ADL at admission), ICU = intensive care unit, infection, LOS = length of stay, eGFR = estimated glomerular filtration rate, MDRD = Modification of Diet in Renal Disease.

**Supplementary Table 3.** Patient characteristics at discharge and post-hospital care.

|  | **Total** | **eGFR_MDRD_ < 45 ml/min/1.73m^2^** | **eGFR_MDRD_ 45-59 ml/min/1.73m^2^** | **eGFR_MDRD_ ≥ 60 ml/min/1.73m^2^** | **N** | ***p-value*** |
| --- | --- | --- | --- | --- | --- | --- |
| **ADL (at discharge)** | 70 (45;85) | 70 (40;80) | 75 (60;90) | 72.5 (45;85) | 425 | 0.004 |
| **Discharge to a nursing home** | 93 (17.1 %) | 43 (28.3 %) | 13 (15.9 %) | 37 (17.1 %) | 453 | 0.014 |
| **Discharge to a nursing home (new)** | 39 (9.8 %) | 20 (15.5 %) | 5 (6.5 %) | 14 (7.3 %) | 399 | 0.028 |

***Notes and abbreviations:*** Data are presented as mean ± standard deviation (SD), number of observations (percentage), or median (IQR = 25^th^;75th percentile). ADL = activities of daily living, eGFR = estimated glomerular filtration rate, MDRD = Modification of Diet in Renal Disease.

**Supplementary Table 4.** Odds of 1-year mortality following fragility fractures by eGFR_MDRD_ levels *without eGFRxSex-interation* (N = 425).

|  | **OR** | **95% CI** | **p-value** |
| --- | --- | --- | --- |
| **Age, years** | 1.00 | 0.94, 1.05 | 0.800 |
| **Female sex** | 1.0 (Ref.) | - | - |
| **Male sex** | 1.37 | 0.69, 2.68 | 0.400 |
| **eGFR (MDRD) categories** |  |  |  |
| **≥60 ml/min** | 1.0 (Ref.) | - | - |
| **45-59 ml/min** | 1.43 | 0.58, 3.39 | 0.400 |
| **< 45 ml/min** | 1.76 | 0.87, 3.60 | 0.120 |
| **ADL Score** | 0.97 | 0.96, 0.98 | **<0.001** |
| **Frailty Index (standardized)** | 1.23 | 0.81, 1.90 | 0.300 |
| **CCI** | 1.16 | 0.98, 1.38 | 0.080 |
| **Fracture Type** |  |  |  |
| **Femur** | 1.0 (Ref.) | - | - |
| **Humerus** | 0.38 | 0.11, 1.08 | 0.100 |
| **Other** | 0.42 | 0.06, 1.59 | 0.300 |
|  | | | |

***Notes and abbreviations:*** Ref. = Reference category for categorical variables, eGFR = estimated glomerular filtration rate (unit: ml/min/1.73m^2^), ADL = activities of daily living, OR = Odds Ratio, CI = confidence interval, MDRD = Modification of Diet in Renal Disease equation, CCI, Charlson Comorbidity Index

**Supplementary Table 5.** Multivariable-adjusted (including heart disease) Hazard Ratios for long-term all-cause mortality and 95% Confidence Intervals.

|  | **HR** | **95% CI** | **p-value** |
| --- | --- | --- | --- |
| Age, years | 1.03 | 1.00, 1.06 | 0.023 |
| Female sex | 1.0 (Ref.) | - | - |
| Male sex | 1.84 | 1.31, 2.59 | <0.001 |
| Frailty Index (standardized) | 1.50 | 1.22, 1.86 | <0.001 |
| **eGFR >= 60 ml/min** | 1.0 (Ref.) | - | - |
| eGFR 45-59 ml/mim | 1.03 | 0.66, 1.62 | 0.9 |
| eGFR < 45 ml/min | 1.85 | 1.30, 2.63 | <0.001 |
| **Fracture Type** |  |  |  |
| Femur | 1.0 (Ref.) | - | - |
| Humerus | 0.94 | 0.60, 1.48 | 0.8 |
| Other | 0.59 | 0.32, 1.08 | 0.088 |
| ADL Score (TV) – spline 1 | 0.98 | 0.96, 1.0 | 0.011 |
| ADL Score (TV) – spline 2 | 1.00 | 0.96, 1.04 | 0.8 |
| CCI (TV) – spline 1 | 0.98 | 0.68, 1.41 | >0.9 |
| CCI (TV) – spline 2 | 0.93 | 0.69, 1.24 | 0.6 |
| CCI (TV) – spline 3 | 0.69 | 0.42, 1.12 | 0.14 |
| Heart disease | 0.84 | 0.60, 1.18 | 0.3 |
|  | | | |

***Notes and abbreviations:*** Ref. = Reference category for categorical variables, SE = standard error, eGFR = estimated glomerular filtration rate (unit: ml/min/1.73m2), ADL = activities of daily living, # OR = Odds Ratio, CI = Confidence Interval, MDRD, HR = Hazard Ratio, CCI, Charlson Comorbidity Index, TV = time varying.

**Supplementary Table 6.** Mediation analyses showing the estimates (with 95% Confidence Intervals) for the effects of impaired kidney function on mortality, before and after accounting for the effect of frailty as a mediator.

| **Effect** | **Estimate (95% CI)** | ***p-value*** |
| --- | --- | --- |
| Total Effect (OR, eGFR ≥ 60 vs < 45) | 1.25 (1.13 - 1.38) | **< 0.001** |
| Average Direct Effect (OR, eGFR ≥ 60 vs < 45) | 1.14 (1.03 - 1.27) | **< 0.001** |
| Average Indirect Effect (eGFR ≥ 60 vs < 45) | 0.1 (0.06 - 0.14) | **< 0.001** |
| Average Proportion Mediated (eGFR ≥ 60 vs < 45) | 0.43 (0.22 – 0.78) | **< 0.001** |
| Total Effect (OR, eGFR ≥ 60 vs 45-59) | 1.02 (0.9 - 1.15) | 0.722 |
| Average Direct Effect (OR, eGFR ≥ 60 vs 45-59) | 0.99 (0.88 - 1.11) | 0.830 |
| Average Indirect Effect (eGFR ≥ 60 vs 45-59) | 0.03 (-0.0 - 0.07) | **0.034** |
| Average Proportion Mediated (eGFR ≥ 60 vs 45-59) | 1.54 (-7.06 – 9.94) | 0.720 |
| Total Effect (OR, eGFR 45-59 vs < 45) | 1.22 (1.07 - 1.4) | **<0.001** |
| Average Direct Effect (OR, eGFR 45-59 vs < 45) | 1.15 (1.01 - 1.31) | **0.04** |
| Average Indirect Effect (eGFR 45-59 vs < 45) | 0.06 (0.02 - 0.1) | **< 0.001** |
| Average Proportion Mediated (eGFR 45-59 vs < 45) | 0.3 (0.12 - 0.93) | **< 0.001** |

**Supplementary Table 7.** Mediation analyses showing the adjusted estimates (with 95% Confidence Intervals) for the effects of impaired kidney function on mortality, before and after accounting for the effect of frailty as a mediator. Models were adjusted for age and sex.

| **Effect** | **Estimate (95% CI)** | ***p-value*** |
| --- | --- | --- |
| Total Effect (OR, eGFR ≥ 60 vs < 45) | 1.21 (1.09 - 1.34) | **< 0.001** |
| Average Direct Effect (OR, eGFR ≥ 60 vs < 45) | 1.12 (1.01 - 1.25) | **0.04** |
| Average Indirect Effect (eGFR ≥ 60 vs < 45) | 0.08 (0.04 - 0.12) | **< 0.001** |
| Average Proportion Mediated (eGFR ≥ 60 vs < 45) | 0.41 (0.21 - 0.92) | **< 0.001** |
| Total Effect (OR, eGFR ≥ 60 vs 45-59) | 1 (0.88 - 1.13) | 0.962 |
| Average Direct Effect (OR, eGFR ≥ 60 vs 45-59) | 0.97 (0.86 - 1.09) | 0.604 |
| Average Indirect Effect (eGFR ≥ 60 vs 45-59) | 0.03 (0 - 0.06) | **0.026** |
| Average Proportion Mediated (eGFR ≥ 60 vs 45-59)* | -15.25 (-11.73 - 6.69) | 0.964 |
| Total Effect (OR, eGFR 45-59 vs < 45) | 1.22 (1.07 - 1.38) | **0.002** |
| Average Direct Effect (OR, eGFR 45-59 vs < 45) | 1.16 (1.02 - 1.32) | **0.022** |
| Average Indirect Effect (eGFR 45-59 vs < 45) | 0.05 (0.02 - 0.09) | **< 0.001** |
| Average Proportion Mediated (eGFR 45-59 vs < 45) | 0.25 (0.09 - 0.74) | **0.002** |

* When the total effect is close to zero, as was the case here (based on a log-odds coefficient of –0.00187, OR = 1), the proportion mediated estimate becomes unstable and may produce inaccurate values, including negative estimates. This instability arises because the proportion mediated is computed as the ratio of the indirect effect to the total effect, and dividing by a near-zero value can amplify random variation. Therefore, for the contrast between eGFR ≥60 and 45–59 mL/min/1.73m², the actual metric should be interpreted as 0 (95% CI: 0 - 6.7, p = 0.964).


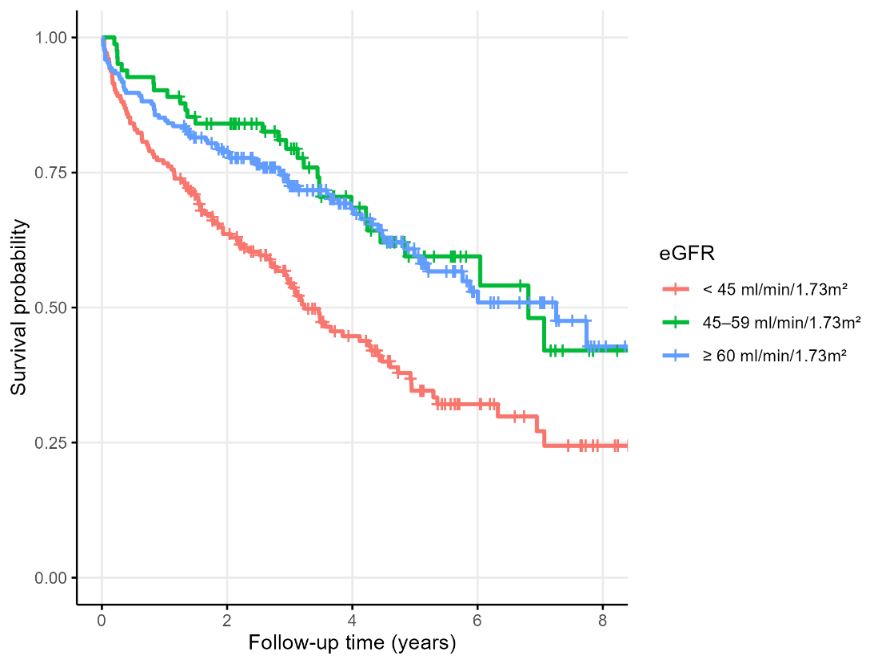


**Supplementary Figure 5.** Kaplan-Meier curves showing survival rates according to eGFR_CKD-EPI_ equation levels. eGFR = estimated glomerular filtration rate, CKD-EPI = Chronic Kidney Disease Epidemiology Collaboration equation.


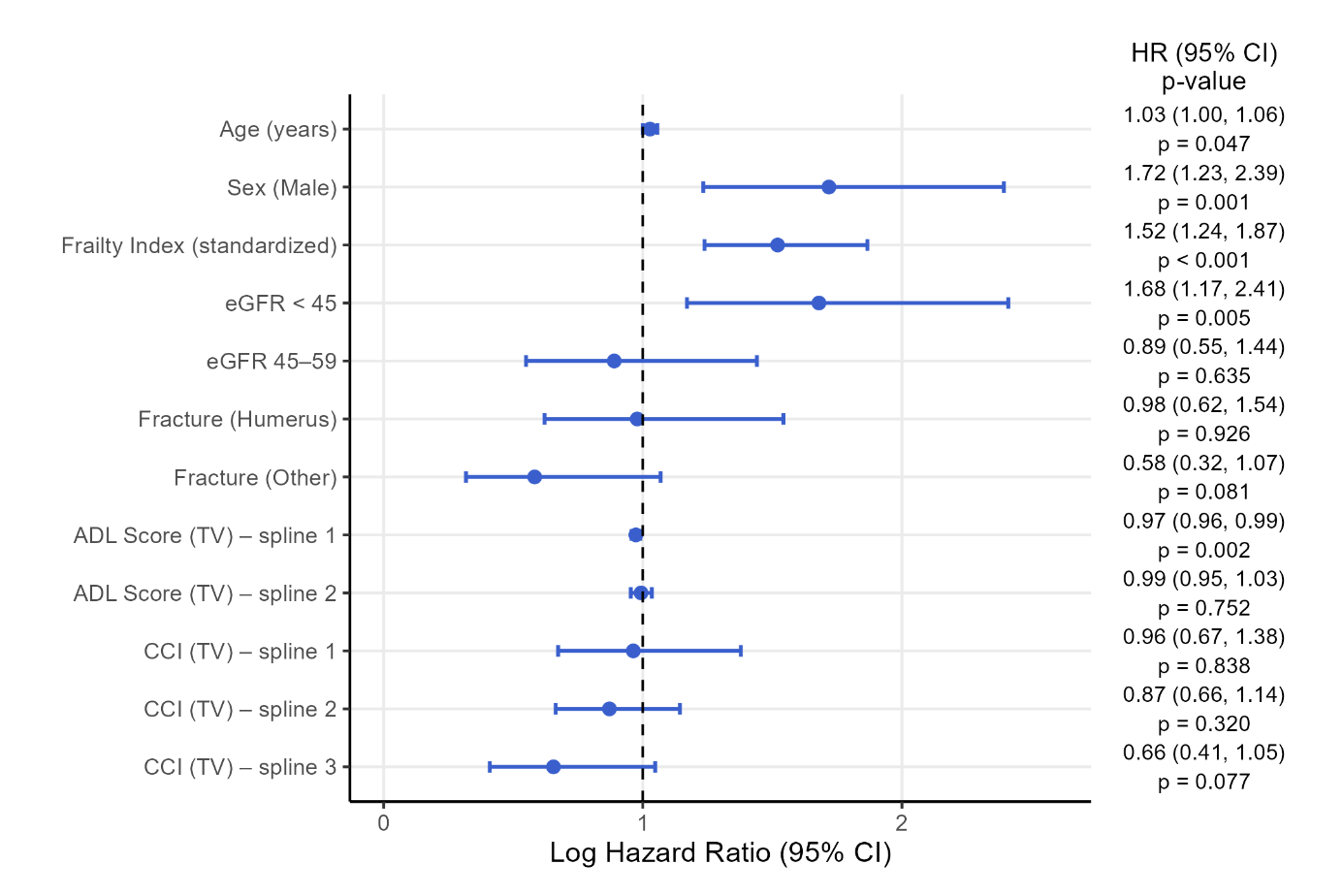


**Supplementary Figure 6.** Forest plot showing the estimates of the Cox regression model for long-term all-cause mortality (log HR, 95% CI) using the eGFR_CKD-EPI_ equation. ADL = activities of daily living, eGFR = estimated glomerular filtration rate, CKD-EPI = Chronic Kidney Disease Epidemiology Collaboration equation, CCI, Charlson Comorbidity Index, TV = time varying.


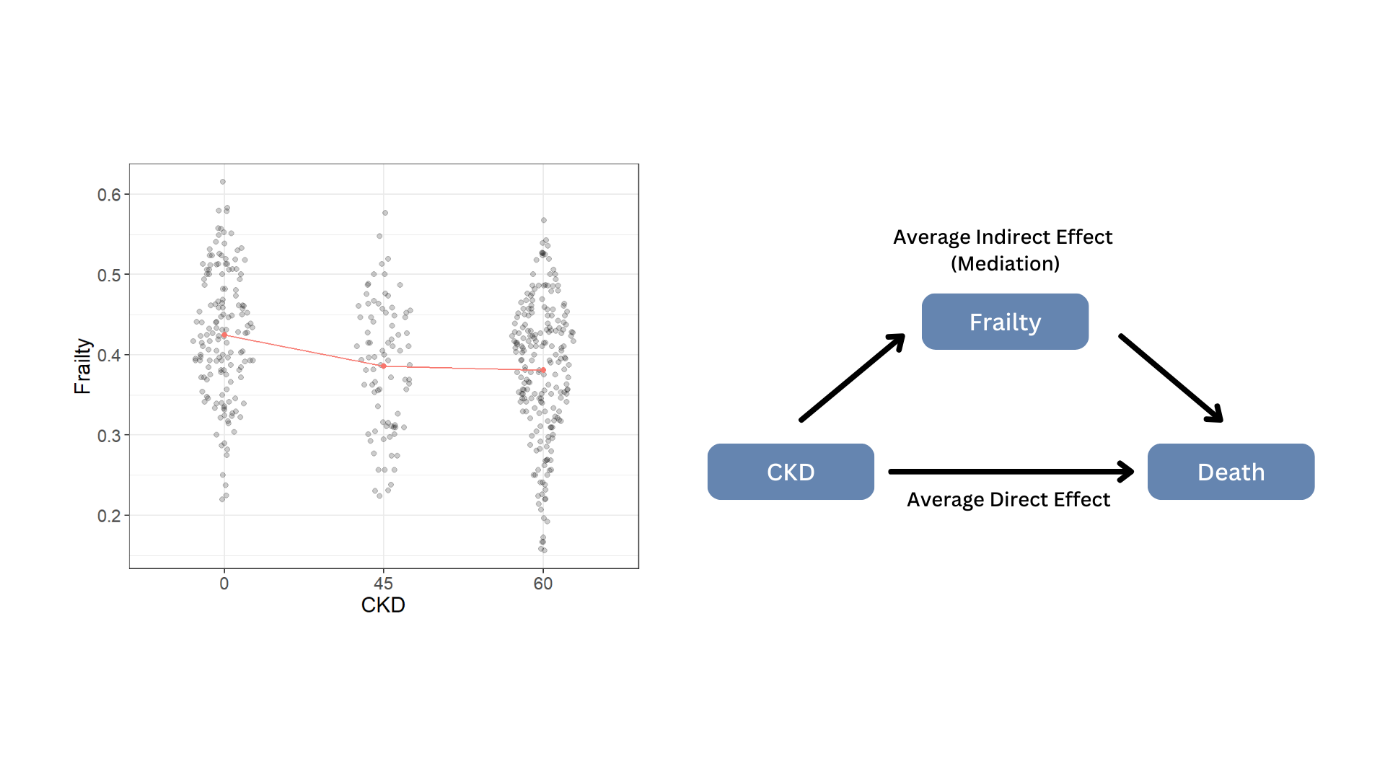


**Supplementary Figure 7.** For the association of impaired kidney function and mortality, potential mediation was explored through frailty, as frailty is a state of increased vulnerability to stressors due to declines in physiological reserves, and it is common in CKD patients due to factors like inflammation, malnutrition, and sarcopenia.


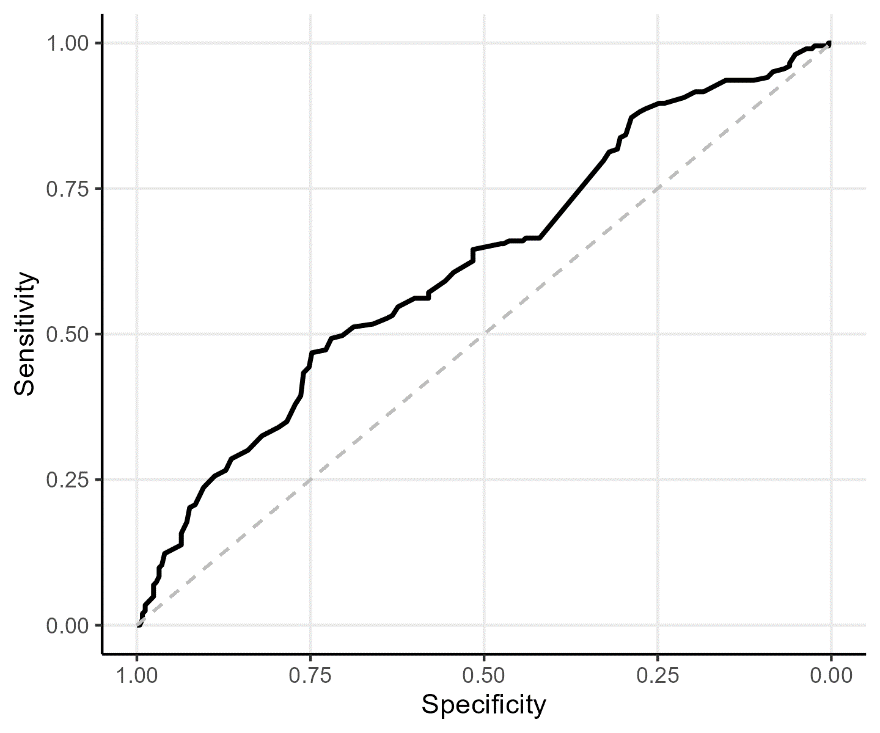


**Supplementary Figure 8.** Receiver Operator Characteristic curve for eGFR_MDRD_, Area Under the Curve = 0.62. eGFR = estimated glomerular filtration rate, MDRD = Modification of Diet in Renal Disease equation.


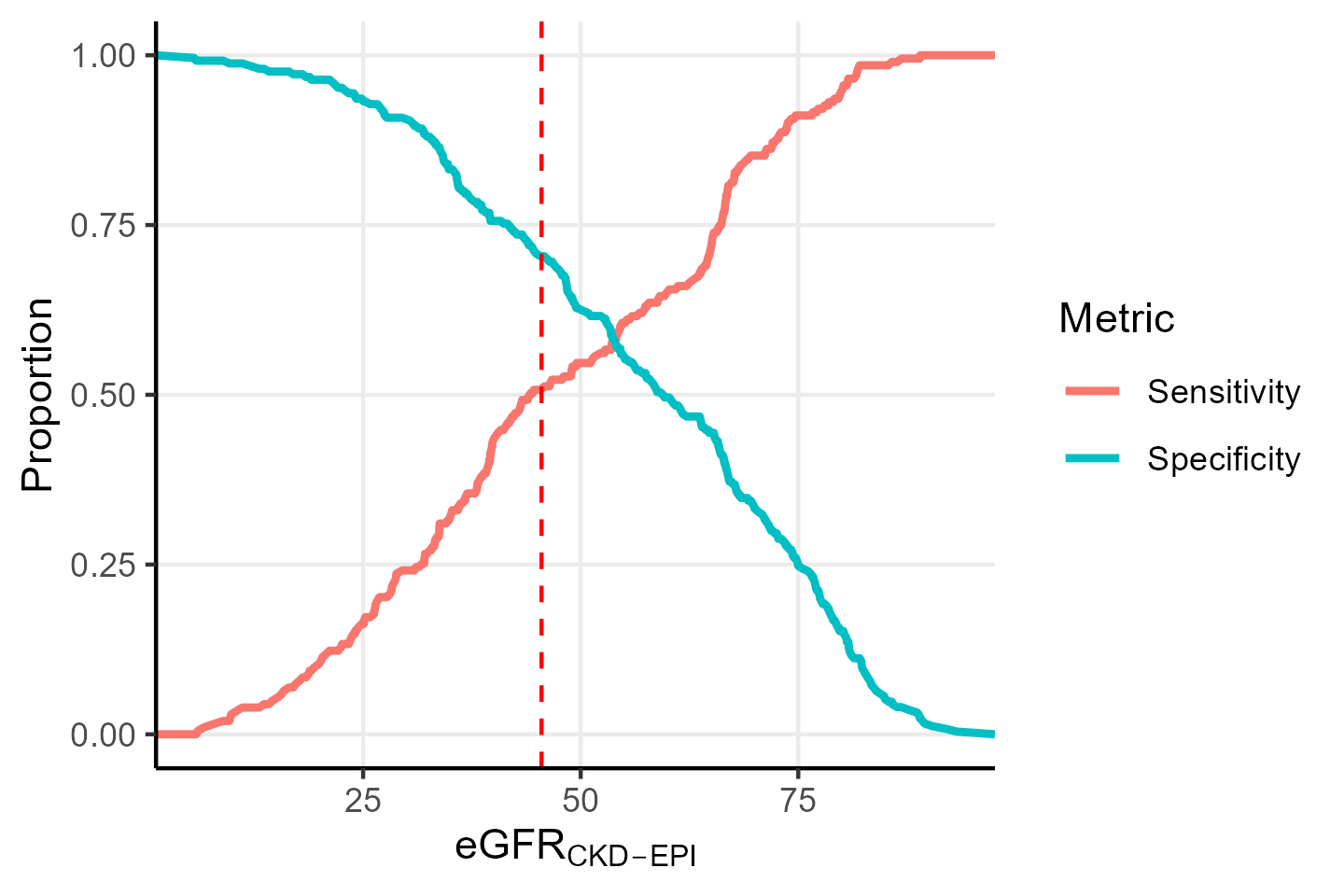


**Supplementary Figure 9.** Sensitivity and specificity as a function of eGFR_CKD-EPI_ values. The eGFR threshold that maximizes the difference between the true positive rate (sensitivity) and the false positive rate (1 - specificity) is shown by the dashed line (eGFR_CKD-EPI_ = 43.3). eGFR = estimated glomerular filtration rate, CKD-EPI = Chronic Kidney Disease Epidemiology Collaboration equation.


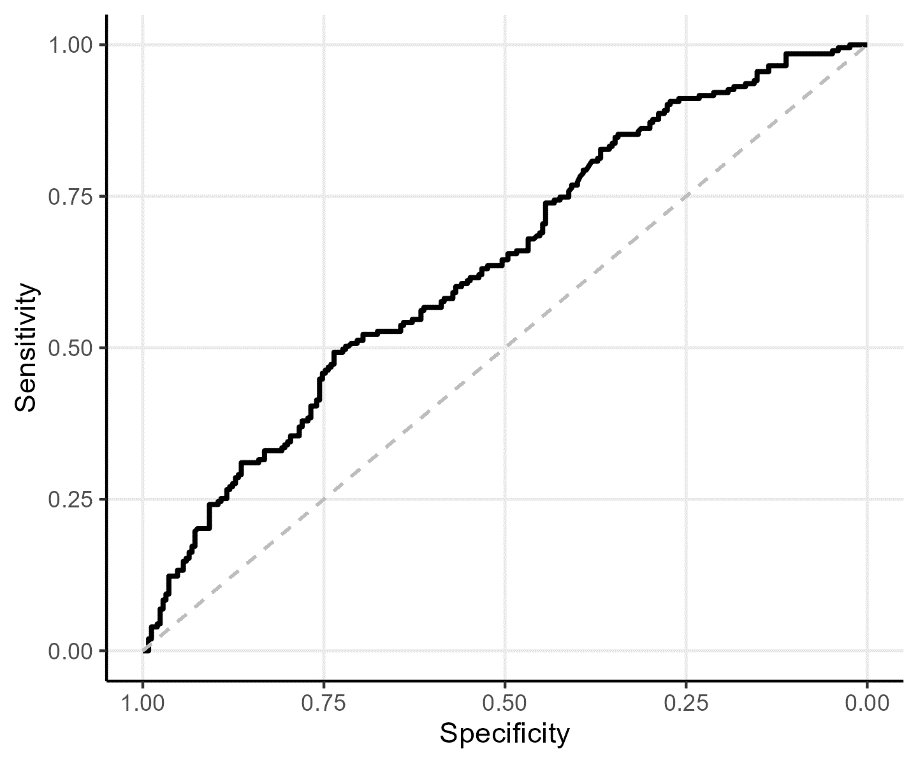


**Supplementary Figure 10.** Receiver Operator Characteristic curve for eGFR_CKD-EPI_, Area Under the Curve = 0.64. eGFR = estimated glomerular filtration rate, CKD-EPI = Chronic Kidney Disease Epidemiology Collaboration equation.
